# Challenging Diagnosis of Lytic Bone Lesions Between Multiple Myeloma and Bone Metastasis of Primary Breast Cancer

**DOI:** 10.7759/cureus.48880

**Published:** 2023-11-16

**Authors:** Tao Edahiro, Hiroshi Ureshino, Tetsumi Yoshida, Noriyasu Fukushima, Tatsuo Ichinohe

**Affiliations:** 1 Hematology and Oncology, Hiroshima University, Research Institute for Radiation Biology and Medicine, Hiroshima, JPN; 2 Department of Internal Medicine, Karatsu Red Cross Hospital, Karatsu, JPN

**Keywords:** computed tomography (ct), missed diagnosis, lytic bone lesion, diagnosis of multiple myeloma, breast cancer metastasis

## Abstract

Lytic bone lesions include various differential diagnoses, such as bone metastasis of cancer, multiple myeloma, primary bone cancers, and infections. Here, we report a rare case of primary breast cancer complicated by lytic bone lesions mimicking bone metastasis, which was subsequently diagnosed as multiple myeloma. Despite the development of several imaging modalities, such as magnetic resonance imaging and positron emission tomography/computed tomography, diagnosing lytic bone lesions with either multiple myeloma or tumor metastasis is highly challenging. Urinalysis is a noninvasive diagnostic method that includes useful diagnostic information; thus, physicians should evaluate urine protein levels when lytic bone lesions are observed.

## Introduction

Breast cancer is the most common newly diagnosed cancer in women [1] and frequently metastasizes to the bone, similar to lung and prostate cancers [2]. Metastatic breast cancer of the bone presents with lytic bone lesions, which include various differential diagnoses, such as multiple myeloma, primary bone cancer, and infections. However, if primary breast cancer is noted, synchronous neoplasms, particularly hematological malignancies (e.g., multiple myeloma), are rarely considered as differential diagnoses of lytic bone lesions because synchronous cancers at the time of the first presentation are rare [3]. We report a rare case of primary breast cancer complicated by lytic bone lesions mimicking bone metastasis, which was subsequently diagnosed as multiple myeloma.

## Case presentation

**Figure 1:**
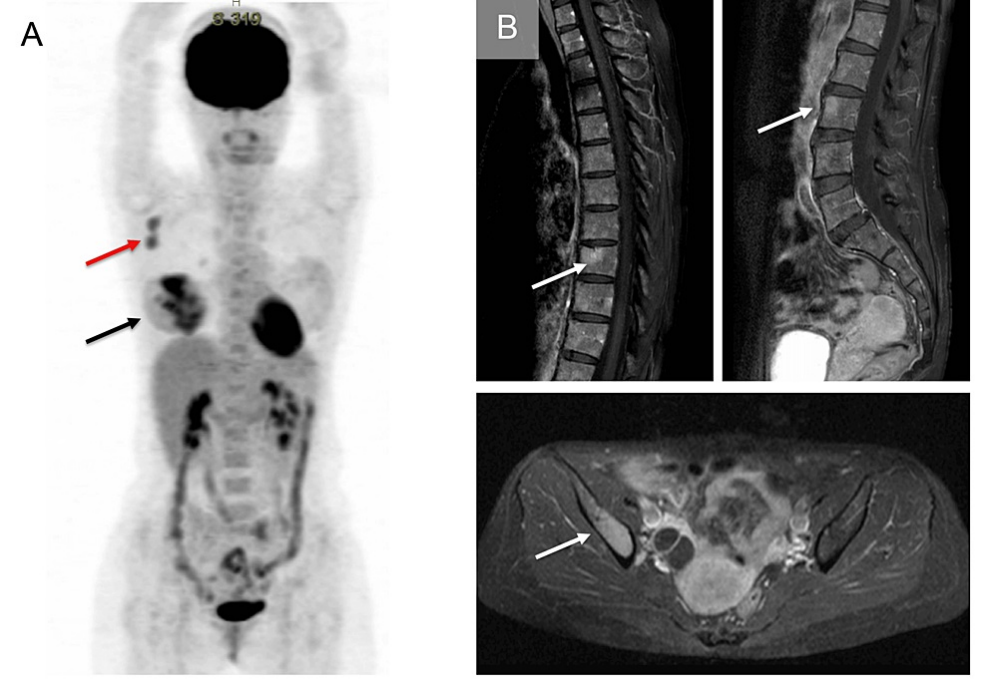
[18F]-fluorodeoxyglucose positron emission tomography/computed tomography [18F]-fluorodeoxyglucose positron emission tomography/computed tomography showing multifocal hypermetabolic right breast lesions (black arrow) with hypermetabolic right axillary lymph nodes (red arrow) (A). T2-weighted imaging with fat suppression magnetic resonance imaging showed multiple high-intensity lesions at the vertebrae and ilium (white arrow)(B).

A 47-year-old woman previously diagnosed with breast cancer (invasive lobular carcinoma) visited our hematology department. Six months before her visit, the patient was diagnosed with right invasive lobular carcinoma by core-needle biopsy. The immunohistochemistry results were as follows: positive for estrogen receptor (ER) (Allred’s total score: 7) and progesterone receptor (PgR) (Allred’s total score: 7) but negative for human epidermal growth factor receptor 2 (HER2). The Ki-67 (MIB-1) labeling index was 29.0% at the hotspot. Then, [18F]-fluorodeoxyglucose (FDG) positron emission tomography/computed tomography (PET/CT) and magnetic resonance imaging (MRI) showed a primary breast lesion, right axillary lymph node metastasis, and multiple lytic bone lesions (rimb, vertebrae, and ilium) (Figure 1A, 1B), indicating multiple bone metastases of primary breast cancer. A sentinel lymph node biopsy was performed, and lymph node metastasis was confirmed. An initial diagnosis of hormone receptor-positive, invasive lobular carcinoma (clinical stage T3N1M1, stage IV) was made; thus, the patient received leuprorelin and tamoxifen. Six months after the initiation of endocrine therapy, primary breast cancer decreased; however, the lytic bone lesions exacerbated. Notably, a new compression fracture was observed. 

**Figure 2:**
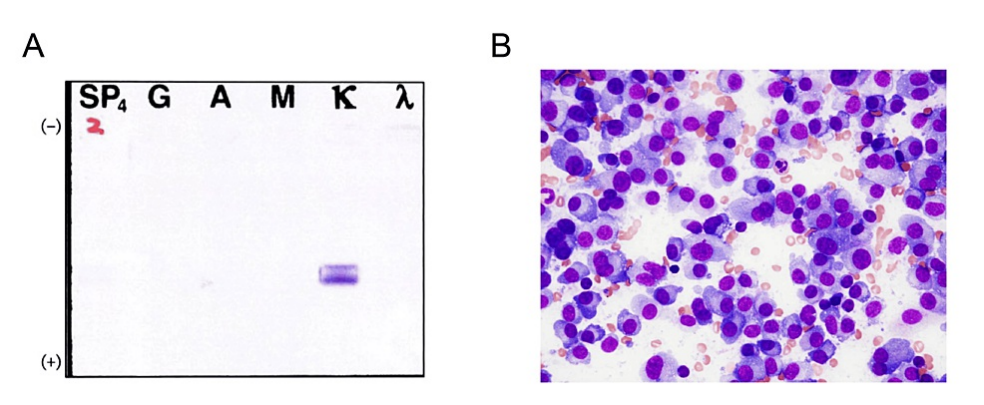
Findings of urine and bone marrow Urine immunofixation showing kappa light chain (A). Bone marrow aspiration revealed increased plasma cells (82%, ×40, May–Giemsa staining) (B).

Laboratory tests revealed slight anemia (hemoglobin of 105 g/L) with normal leukocyte (4.7 × 109/L) and platelet (264 × 109/L) counts. The biochemical test revealed normal total protein (73 g/L) and albumin (46 g/L) levels and decreased immunoglobulin A (IgA) (0.6 g/L) and IgM (0.3 g/L) levels with normal IgG levels (9.5 g/L). The patient’s serum creatinine level was normal (7.3 mg/L), whereas high urine protein levels were detected (7.8 g/day). The Bence-Jones protein (kappa chain) was detected using urine immunofixation electrophoresis (Figure 2A). Serum kappa free light chain levels were extremely elevated (38 g/L), lactate dehydrogenase level was normal (207 U/L), β2 microglobulin level was slightly elevated (2.5 mg/L), and serum calcium level (98 mg/L) and carcinoembryonic antigen (1.7 ng/mL) and cancer antigen 15-3 (9.3 U/mL) levels were normal. Bone marrow aspiration from posterior iliac crest revealed increased plasma cells (82%) (Figure 2B), suggesting the existence of multiple myeloma. G-banded metaphase analysis revealed a normal karyotype, and fluorescence in situ hybridization revealed no high-risk cytogenetic abnormalities, including t(4;14), t(14;16), and del(17p). Multiple myeloma, Revised International Staging System stage I, was diagnosed; therefore, the lytic bone lesions were not defined as breast cancer metastasis.

Subsequently, right radical mastectomy with axillary lymph node dissection was performed. The final pathological diagnosis was invasive lobular carcinoma, with right axial lymph node metastases, pathological stage, pT3 (55 × 48 mm), pN1 (3/17 positive), pM0, stage IIIA. The immunohistochemistry results were as follows: positive for ER (Allred’s total score: 6) and PgR (Allred’s total score: 3) but negative for HER2. The Ki-67 (MIB-1) labeling index was 1.0%.

**Figure 3:**
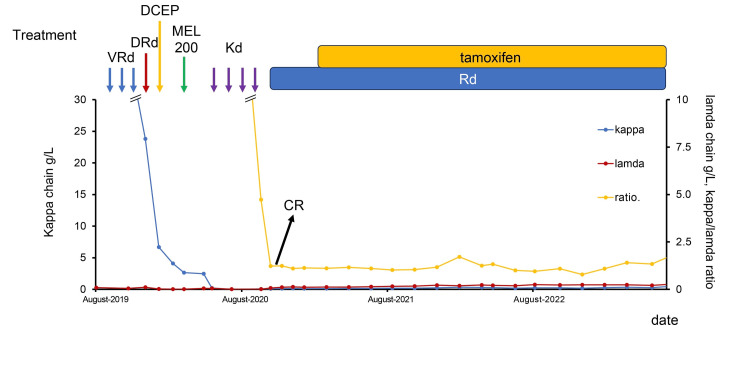
Clinical course VRd, bortezomib, lenalidomide, and dexamethasone; DRd, daratumumab, lenalidomide, and dexamethasone; DCEP, dexamethasone, cyclophosphamide, etoposide, and cisplatin; MEL, melphalan; Kd, carfilzomib and dexamethasone; CR, complete response

Four weeks after the surgery, the patient received bortezomib, lenalidomide, and dexamethasone (VRd); subsequently, tamoxifen and denosumab were initiated. Denosumab was also initiated for bone lesions. One week after the initiation of tamoxifen, grade 3 liver damage occurred; thus, tamoxifen was discontinued, decreasing the patient’s transaminase levels. She achieved partial response (PR) after three cycles of VRd. Because daratumumab can improve treatment response depth [4], we changed the treatment regimen to daratumumab, lenalidomide, and dexamethasone (DRd); then, peripheral blood stem cells were collected using dexamethasone, cyclophosphamide, etoposide, and cisplatin followed by granulocyte colony-stimulating factor [5]. Autologous peripheral blood stem cell transplantation (PBSCT) (infusing 2.7 × 106/kg of CD34+ cells) following high-dose melphalan was performed, and the patient achieved PR. Four posttransplant courses of consolidation therapy with carfilzomib and dexamethasone (Kd) yielded complete response (CR). Maintenance therapy with lenalidomide and dexamethasone (Rd) was initiated, and then, tamoxifen was resumed four months after the initiation of Rd. Tamoxifen did not exhibit any severe adverse events (particularly liver damage) at that time. Rd and tamoxifen were continued to date, and no disease relapse of both multiple myeloma and breast cancer was observed 52 months after autologous PBSCT (Figure 3).

## Discussion

We report a rare coincident case of primary breast cancer and multiple myeloma. In this case, lytic bone lesions were initially misdiagnosed as primary breast cancer metastasis; thus, the case implies an important pitfall in making differential diagnoses for lytic bone lesions. Synchronous neoplasms, coincident with breast cancer and multiple myeloma, are rare; indeed, only a few cases have been reported [6-8]. Meanwhile, breast cancer is the most frequent cancer in women, and the incidence of multiple myeloma has recently increased [9]. Thus, the coincidence of myeloma and breast cancer is expected to increase.

The common metastatic site for breast cancer is the bone, and patients with large primary tumors and positive axillary lymph node metastasis [10,11] are at a high risk of bone metastasis. Therefore, the patient in this case report was considered to be at a high risk of bone metastasis. Both denosumab (monoclonal antibody against receptor activator of nuclear factor κB ligand) and zoledronic acid (bisphosphonate) are effective in preventing skeletal-related events in patients with both bone metastases of breast cancer [12] and multiple myeloma [13].

Diagnosing lytic bone lesions with either multiple myeloma or tumor metastasis is highly challenging. Although MRI and FDG PET/CT have been reported to be useful tools in distinguishing multiple myeloma from cancer metastasis involving the spinal bone [14], various evaluative methods using these tools have been investigated; thus, a standard imaging method has not yet been established. Meanwhile, the quantification of M-protein in the serum or urine is valuable in diagnosing multiple myeloma [15]. In the patient in this case report, elevated urine protein levels and the presence of urine Bence-Jones protein helped us make an accurate diagnosis. Notably, patients with stage IIIA and IV breast cancer have distinct survival outcomes [16], and survival outcomes in patients with multiple myeloma were improved by introducing new drugs [17]. Thus, making an accurate diagnosis is necessary.

## Conclusions

The diagnosis of lytic bone lesions is challenging. A standard imaging method including MRI or FDG PET-CT has not been established. We recommend that physicians should evaluate urine protein levels when lytic bone lesions are noted because urinalysis is a noninvasive evaluation method that includes useful diagnostic information. The evaluation of the M-protein is valuable in diagnosing multiple myeloma.
